# eDNA-based monitoring of parasitic plant (*Sapria himalayana*)

**DOI:** 10.1038/s41598-019-45647-5

**Published:** 2019-06-24

**Authors:** Maslin Osathanunkul

**Affiliations:** 1 Department of Biology, Faculty of Science, Chiang Mai University, Chiang Mai, 50200 Thailand; 2 Center of Excellence in Bioresources for Agriculture, Industry and Medicine, Department of Biology, Faculty of Science, Chiang Mai University, Chiang Mai, Thailand

**Keywords:** Environmental biotechnology, Environmental sciences

## Abstract

*Sapria himalayana* Griffith., is a root parasitic plant that is exceptionally beautiful and odd-looking and found in Southeast Asia. Now these plants are at risk of extinction as they face a large number of different threats. Appropriate measures and conservation plans are needed and one crucial key for successful conservation is species monitoring. The flower is the only part of *S. himalayana* that is visible during a short period of time of the year. Thus, conducting a visual survey in the field at the other times of the year would be difficult. DNA from living organisms could be found accumulating in environment and so-called environmental DNA (eDNA). Here, an eDNA-based method was developed to specifically monitor *S. himalayana* in nature. Detecting the specifically generated amplicons allowed us to monitor the presence of *S. himalayana* at any time of the year. This developed method would increase the conservation success of the *S. himalayana*.

## Introduction

Angiosperms are a taxonomically diverse group of plants that include parasitic plants. Parasitic plants rely on host plants for water and nutrients. Parasitic plants may be classified in various ways. However, the most common way is based on either their point of attachment to the host (shoot or root parasites) or, the absence or presence of chlorophyll (hemiparasites or holoparasites, respectively)^1^. Rafflesiaceae are nonphotosynthetic parasites, which are leafless, stemless, and rootless and depend on their host plants for nutrition^2,3^. Plants in Rafflesiaceae, are exceptionally beautiful aesthetically and odd-looking. This family includes the genera *Rafflesia* (28 species), *Rhizanthes* (4 species), and *Sapria* (3 species). Rafflesiaceae are found in tropical rainforests of Southeast Asia. The three species of *Sapria* were recorded in sub-tropical regions of mountain forests in the Southeast Asia^4,5^. Three *Sapria* species (*Sapria himalayana* Griffith., *Sapria poilanei* Gagnep. and *Sapria ram* Bänziger & B. Hansen) have been recorded in Thailand. *Sapria* understory plants, live underground for most of their lives and during a specific period, small protuberances emerge from the roots or near-ground stems of the vine *Tetrastigma* (Vitaceae). The flowers are about 20 cm in size and unisexual. They have 10 bright red with sulphur-yellow spots bracts. They are at the brink of extinction due to both their nature and incessant human intervention in the natural forest environment.

**Figure 1:**
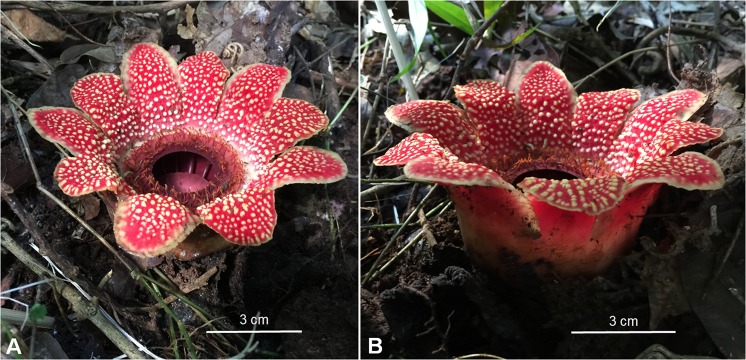
Flowers of *S. himalayana* found at the studied site in the Doi Suthep-Pui National Park, Chiang Mai, Thailand (**A**,**B**).

Hermit’s spittoon, *S. himalayana* (Rafflesiaceae) is a root parasitic plant. It is one of the lesser-known taxons which can be found in the north of Thailand (Fig. 1). *S. himalayana* species have been found to parasitise on roots of *Tetrastigma* species (*T. obovatum, T. laoticum* and *T. cruciatum*)^5^. With a preference for specific hosts, they lose their chance to survive if their host plant is removed or destroyed. Habitat fragmentation and habitat loss results from ever-expanding human population. This constantly requires additional resources and space and thus opens up new settlements. Intensive agriculture to meet people’s demands and several other human activities contribute to cause *S. himalayana* to become rare or threatened with extinction. In addition, *S. himalayana* has a naturally high bud mortality at 46–67% and low fruiting rate as little as 8–12%^6^. Their chances of survival are so small that appropriate measures and conservation plans are needed so that they have a better chance to thrive. One crucial key for successful conservation is species survey and monitoring. However, most of this species’ life is spent underground and the only part of the plant that emerges from host is the flower. Their flowers are usually visible during a specific time of the year and thus, monitoring the *S. himalayana* at the other times of the year would be difficult. Seasonal activity or behaviour has an impact on detection probabilities and a further huge impact on conventional field sampling approaches to measure the abundance or absence/presence of organisms^7,8^. Reliable survey methods for monitoring parasitic plants that could minimise such impacts would greatly advance assessment and monitoring and thus make for effective conservation. Physical and/or visual detection rates for root parasitic plant species vary substantially between sites, species and time of the year as detecting those parasitic plants would be only possible after their flowers emerge. Currently applied DNA-based methods work reasonably well to enumerate species for natural resource management and conservation. Advanced molecular techniques facilitate the estimating and monitoring of biodiversity, especially the increasing application of environmental DNA or eDNA. This has proven to be a sensitive, effective and convenient method with increased speed^9–11^. DNA from living organisms including animals, plants and fungi could be found to accumulate in environment and so-called environmental DNA (eDNA)^12,13^. Using eDNA for species monitoring is performed by detecting DNA fragments that organisms of interest release into the environments. DNA found in environments could originate from various sources. The use of eDNA for species monitoring and detection is becoming more popular, with an increasing number of studies dedicated to both testing and applying these methods. However, these are mainly applied to aquatic organisms, including various fish, amphibians, and mammals^11,14–16^. To date, no study has used eDNA for parasitic plants.

In the present study, the eDNA-based method for monitoring root parasitic plants species using hermit’s spittoon (*S. himalayana*) as a model species has been developed and tested. Species-specific primer pairs that amplify DNA fragments only in *S. himalayana* were used to detect species in combination with quantitative real-time PCR (qPCR). An eDNA detection method from soil for *S. himalayana* was established first. Next, distribution surveys were conducted based on eDNA for this species in the Doi Suthep-Pui National Park, Chiang Mai, Thailand when both the flowers of *S. himalayana* were visible and no visible trace remained above-ground. The results were compared to visual observation during the time when its flowers could be spotted in order to prove that the adapted protocols, could be used to detect *S. himalayana* eDNA in both aboveground and belowground stages of life.

## Results

*S. himalayana* DNA was successfully amplified in soil samples taken from the sites where we spotted the *S. himalayana* buds and the host *Tetrastigma* species (SSH1-SSH3). The target species was detected in all three sampling sites. No amplified DNA was found in samples from where *S. himalayana* was not observed (NSH and SBF), in all three replicates, indicating that there is consistency in detection of the target species. Similarly, there was no amplification of the target species in all replicates observed from negative samples (Table 1).

**Table 1:** Detection of Hermit’s spittoon DNA using qPCR (Raw data are shown in Table S2).

Location/Dilution	Visual detection		November 2017	eDNA detection*	
	October 2016	April 2017		Positive qPCRs replicates	Cycle threshold mean, range
Positive control (DNA extracted from tissue)	—	—	—	3/3	14, 13–14
1/10 dilution	—	—	—	3/3	17, 17
1/10^2^ dilution	—	—	—	3/3	21, 21–22
1/10^3^ dilution	—	—	—	3/3	25, 24–25
1/10^4^ dilution	—	—	—	3/3	26, 26–27
1/10^5^ dilution	—	—	—	3/3	28, 28
SSH1	Yes	no	yes	3/3	15, 15–16
SSH2	yes	no	yes	3/3	23, 23
SSH3	yes	no	yes	3/3	24, 24–26
~1 m from bud/flower	no	no	no	3/3	30, 29–31
~5 m from bud/flower	no	no	no	2/3	33, 32–34
NSH	no	no	no	0/3	—
SBF	no	no	no	0/3	—

**Figure 2:**
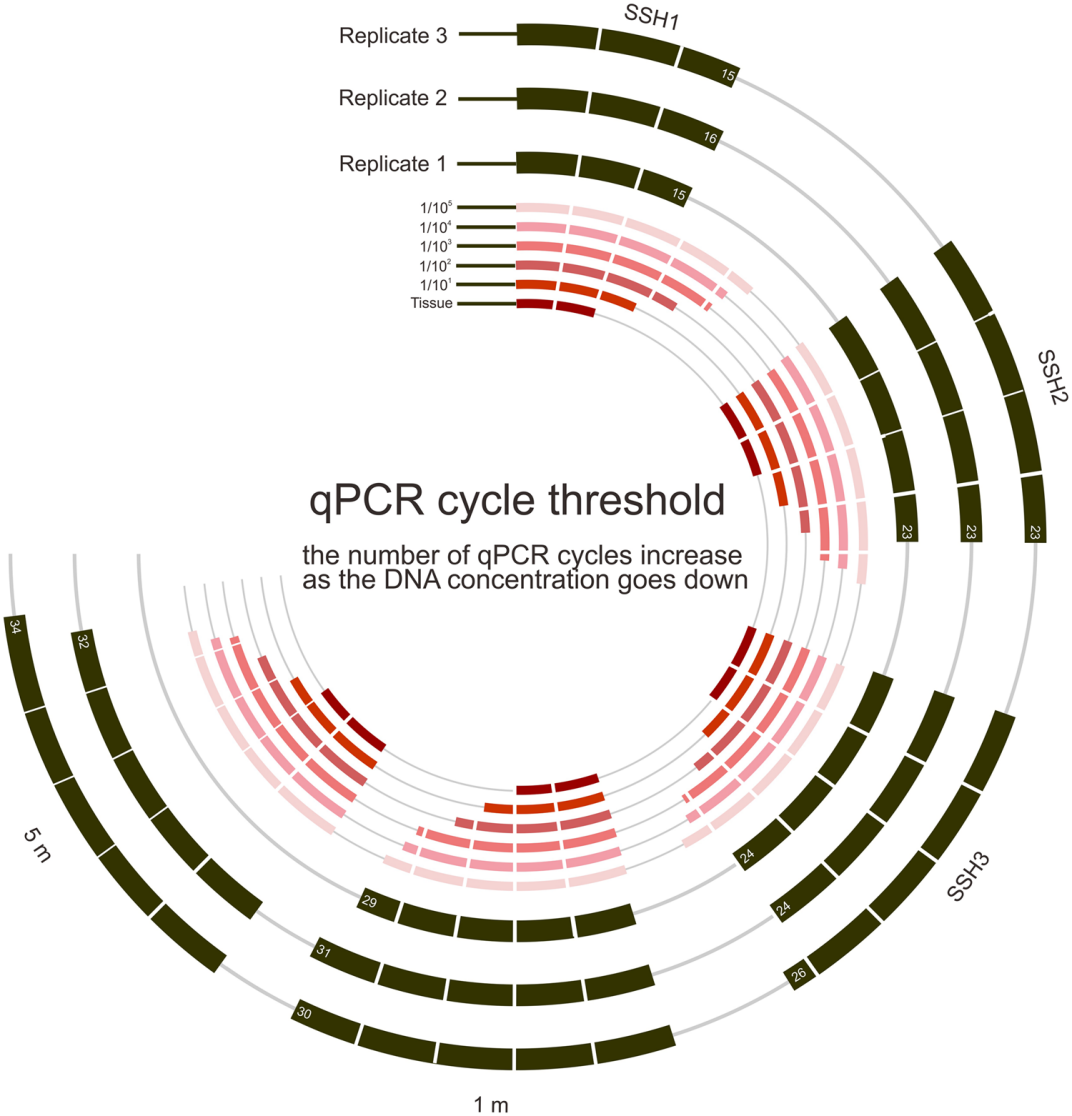
qPCR cycles required for eDNA detection from soil. qPCR cycle threshold from three replicates comparing between sites and *S. himalayana* DNA dilution series (1/10^1^–1/10^5^).

Except for the negative control sites (NSH and SBF), the qPCR results detected the *S. himalayana* within all soil samples (Table 1). The sequences of the amplicons were a 100% match with both the reference sequences from tissue samples of the *S. himalayana* and from GenBank. The number of cycles in qPCR analysis which was required for detection was also consistently between 15–16 cycles for all eDNA triplicates of the SSH1 soil sample, this was slightly different from the value obtained from the tissue-derived DNA (13–14 cycles) (Table 1). With optimal PCR efficiency, this indicates a minimal difference of around 1 order of magnitude in DNA concentration. Additionally, the *S. himalayana* DNA was successfully amplified in all three replicates on individual soil samples SSH2 and SSH3 with 23–24 cycles (difference of ~2–3 orders of magnitude in DNA concentration) (Fig. 2). *S. himalayana* eDNA was also amplified in two out of three qPCRs from soil samples collected at a distance of within 5 m from the buds/flowers (Table 1) with 32 and 34 qPCR cycles, as expected for eDNA extracted from diluted environmental samples. No positive PCRs were obtained from DNA extracts from NSH and SBF, where there is no record of hermit’s spittoon and its host.

## Discussion

eDNA of *S. himalayana* extracted from soil was successfully detected in all sites where *S. himalayana* and hosts were recorded. To the extent of our knowledge, this is the first work on eDNA detection of root parasitic plants in both aboveground and belowground stages of life. The results suggest that it is possible to use eDNA in soil samples to detect presence of parasitic plants and thus could have potential use in future conservation plans/management.

The DNA dilution series (1/10^1^–1/10^5^) was used in qPCRs and found that the number of qPCR cycles increased as the DNA concentration went down (Table 1 and Fig. 2). Comparing the required number of cycles in qPCR analysis for detection at the SSH1site with the DNA from tissue would indicate a minimal difference of around 1 in the order of magnitude in DNA concentration which is unsurprising as we collected the soil samples directly from the *S. himalayana* buds/flowers. Whereas, the required number of qPCR cycles at the site around 5 m away from the buds/flowers indicates a minimal difference of around 4–5 orders of magnitude in DNA concentration. One of three PCRs was found to be negative in the soil sample collected from around 5 m away from the buds/flowers. This could be a result of diluted environmental samples rather than a false negative. While, in water sampling, increasing the volume of sample may reduce the rate of false negatives (e.g.^11^), there is no such report in soil sampling.

It can be seen that the required number of qPCR cycles for SSH2 detection (23 cycles) and SSH3 (24 cycles) were similar with a DNA concentration of around 1/10^3^. Although, when the soil samples were collected in October 2016, the flowers had already emerged and could be seen on the ground and thus, differences in the flower stages between sites were observed. At the SSH2 and SSH3 sites, most of them were still buds (globose with white and pink bracts), while all *S. himalayana* spotted at the SSH1 site were either in bloom or the flowers dehisced and became dark in colour. The differences in flower stages may lead to the difference of qPCR cycles required for detection in SSH1, SSH2 and SSH3 sites. Thus, further investigation would be interesting and should be carried out to better understand the matter.

The molecular approach, based on eDNA extraction from soil, is already commonly used to characterise soil microorganisms and its application is now being used to characterise other soil organisms^17^. eDNA detection is conducted in a variety of environments such as agricultural fields, deserts, forests, the Arctic and Antarctic^18,19^. The availability of an eDNA-based method will provide new options for monitoring and surveying root parasitic plants. Applying the method for detection of root parasitic weeds will be also useful. This can refer to detecting parasitic weeds such as *Striga* and *Orobanche* spp., which are difficult to control as their life cycles are mainly underground. This leads to difficulty diagnosing infection already done before the parasites emerge^20^. The method can be also used to detect other rare and economically parasitic plants such as *Rafflesia* and *Balanophora*.

## Methods

### Floral and soil materials and DNA extraction

*S. himalayana* buds were collected at Doi Suthep-Pui National Park, Chiang Mai, Thailand. Soil samples were collected from three different sites and also at Doi Suthep-Pui National Park, Chiang Mai, Thailand. Soil samples came from the following key areas: where we spotted the *S. himalayana* buds and the host *Tetrastigma* species (called SSH), from where we did not see both buds and host (called NSH), and from where we found another parasitic plant, *Balanophora fungosa* J. R. Forst. & G. Forst. (called SBF). Three triplets of soil samples were taken per site in October 2016 (when flowers were emerging) and again in April 2017 (when there were no flowers). In November 2017, the studied sites were visited for visual detection only and no further soil samples were collected.

All three soil samples per site were pooled, mixed and air-dried at 30–40 °C for 24–48 h. Dried soil was ground into fine powder which was subsequently used for the DNA extraction^21^. DNA was extracted from 500 mg of soil per sample using the NucleoSpin® Soil Kit (Macherey Nagel™) according to the manufacurer’s protocols. Each pooled sample was extracted in triplicates and then ready to be used in next analysis.

### Species-specific primers designing

To design primers specific to *S. himalayana*, we sequenced the partial ITS region from three individuals of each *S. himalayana* and *B. fungosa*, which is the closest related parasitic plant to our target species in the studied area. *S. himalayana* and *B. fungosa* buds/flowers were collected at Doi Suthep-Pui National Park, Chiang Mai, Thailand. The total DNA was extracted from tissue samples using the Nucleospin Plant II kit (Macherey-Nagel, Germany) according to the manufacturer’s protocol. From searching through the public databases, there was no available sequence of the parasitic plants species collected from the studied area. We therefore amplified and sequenced the partial ITS regions with the primers B330F 5′ TGACGGGTGACGGAGAATTAGG 3′ and B1764R 5′ CAATAATCCTTCCGCAGGTTCACC 3′, both of which were modified from previous sequences of parasitic plants retrieved from GenBank (Table S1). For qPCR analysis, species specific primers Sapria_ITSF 5′ TGTCGGATTTTCCGTCTCATCC 3′ and Sapria_ITSR 5′ GTCACACGATTAATCGCTCGTACA3′ were designed using the PrimerBlast software (http://www.ncbi.nlm.nih.gov/tools/primer-blast/) to target a short (191 bp) fragment of the ITS region of *S. himalayana* using sequences from the consensus sequence generated by this work (Table S1) and also GenBank and checked against all other *S. himalayana* sequences in GenBank at the time.

The specificity of the primers was tested by comparing the sequences to other *Sapria* species including *B. fungosa* species which had been found in the studied area. The designed primers contain no less than 5 mismatches with non-target species.

### qPCR analysis

The qPCR was conducted in a 20 μL reaction volume containing 10 μL of ABI TaqMan Universal Master Mix II, 1 μL of each primer, and 2 μL of DNA extract. The qPCR conditions were as follows: 10 min at 95 °C and 50 cycles of 30 s at 95 °C, and 45 s at 51 °C, and 30 s at 72 °C. All samples were run as triplicates. Amplifications were conducted using the Rotor-Gene Q System (Qiagen, Germany), and Cq values were automatically set using the system software. In all qPCR, the R^2^ values for the standard curve were ≥0.97 and efficiency 90–97%.

Three replicates in each qPCR set contained only reagents but no DNA were used as a negative control whilst one tube that contained all reagents and *S. himalayana* extracted DNA template was used as a positive control. The absence/presence calls was determined from data of the post-PCR read. Only the target amplified above the target’s threshold obtained from the default analysis settings in the Rotor-Gene Q software version 2.3.1 (Qiagen, Germany), in the target species’ eDNA was called present^22^.

## Supplementary information

Table S1

Table S2
