# Supplementary material for: eDNA-based monitoring of parasitic plant (Sapria himalayana)
Source: Sci Rep. 2019 Jun 24;9:9161. doi: 10.1038/s41598-019-45647-5 (PMC6591406; doi:10.1038/s41598-019-45647-5)
Supplement: Supplementary file 1 — Table S1 [file 41598_2019_45647_MOESM1_ESM.pdf]

eDNA-based monitoring of parasitic plant (*Sapria himalayana*)

Maslin Osathanunkul[1,2*]

[1]Department of Biology, Faculty of Science, Chiang Mai University, Chiang Mai, 50200, Thailand

[2]Center of Excellence in Bioresources for Agriculture, Industry and Medicine, Chiang Mai University

Table S1: Genebank accessions numbers of previously published sequences and own generated sequences used in this study.

Scientific Name	Genebank Accession Number
Rafflesia keithii	AY739084
Rafflesia pricei	AY739083
Rhizanthes infanticida	L24048
Rhizanthes lowii	FJ669712
Sapria himalayana	MG969941*
Sapria himalayana	MG969942*
Sapria himalayana	AY739086
Sapria poilanei	AY739087
Sapria ram	AY739088

* own generated sequences
