# Supplementary material for: eDNA-based monitoring of parasitic plant (Sapria himalayana)
Source: Sci Rep. 2019 Jun 24;9:9161. doi: 10.1038/s41598-019-45647-5 (PMC6591406; doi:10.1038/s41598-019-45647-5)
Supplement: Supplementary file 2 — Table S2 [file 41598_2019_45647_MOESM2_ESM.pdf]

eDNA-based monitoring of parasitic plant (*Sapria himalayana*)

Maslin Osathanunkul[1,2*]

[1]Department of Biology, Faculty of Science, Chiang Mai University, Chiang Mai, 50200, Thailand

[2]Center of Excellence in Bioresources for Agriculture, Industry and Medicine, Chiang Mai University

Table S2: qPCR raw data generated in this study.

Excel Analysed Data Export
Copyright (c) 2013 QIAGEN GmbH. All Rights Reserved.
File Specific Sapria 51C sampleOCT A.rex

Operator	MO
Run Id	
Notes	
Machine Serial No	814137

Channel	Gain
Green	5
Yellow	5
Orange	5
Red	5
Crimson	7
HRM	7

Channel	Threshold
Relative Quant. Analysis	
Cycling A.Green (Page 1)	0.05574

Quantitative analysis of Cycling A.Green (Page 1)

No.	Color	Name	Type	Ct	Ct Comment	Given Conc (Copies)	Calc Conc	% Var	Rep. Ct	Rep. Ct Std. Dev.	Rep. Ct (95% CI)	Rep. Calc. Conc.	Rep. Calc. Conc. (95% CI)
1	255	Sapria 1/10	Standard	16.94		0.1	1.11E-01		16.81			1.28E-01	
2	8388736	Sapria 1/10	Standard	16.88		0.1	1.27E-01						
3	14450322	Sapria 1/10	Standard	16.63		0.1	1.47E-01						
4	32896	Sapria 1/100	Standard	21.08		0.01	1.26E-02		21.11			1.24E-02	
5	16711680	Sapria 1/100	Standard	21.34		0.01	1.11E-02						
6	11433472	Sapria 1/100	Standard	20.92		0.01	1.36E-02						
7	11403264	Sapria 1/1000	Standard	24.29		0.001	1.21E-03		24.17			1.25E-03	

8	11338136	Sapria 1/1000	Standard	24.07		0.001	1.23E-03		
9	6553774	Sapria 1/1000	Standard	24.16		0.001	1.31E-03		
10	14013909	Sapria 1/10000	Standard	26.79		0.0001	1.17E-04	26.8	1.16E-04
11	12566463	Sapria 1/10000	Standard	26.58		0.0001	1.26E-04		
12	9803157	Sapria 1/10000	Standard	27.05		0.0001	1.06E-04		
13	7697781	Sapria 1/100000	Standard	28.09		1E-05	1.62E-05	28.27	1.60E-05
14	5526612	Sapria 1/100000	Standard	28.25		1E-05	1.61E-05		
15	255	Sapria 1/100000	Standard	28.48		1E-05	1.58E-05		
16	51400	Sapria tissue	Unknown	12.97			5.38E+00	13.63	4.67E+00
17	32768	Sapria tissue	Unknown	14.44			3.80E+00		
18	65280	Sapria tissue	Unknown	13.48			4.83E+00		
19	65280	1m	Unknown	30.87			1.30E-05	30.22	1.38E-05
20	65408	1m	Unknown	29.76			1.43E-05		
21	65408	1m	Unknown	30.04			1.40E-05		
22	8454016	5m	Unknown	32.16			1.15E-05	33.34	1.01E-05
23	8454016	5m	Unknown	34.53			8.77E-06		
24	4259584	5m	Unknown						
25	4259584	site1	Unknown	15.92			1.47E-02	15.97	1.53E-01
26	8388608	site1	Unknown	16.04			2.16E-01		
27	8388608	site1	Unknown	15.95			2.27E-01		
28	16711680	site2	Unknown	23.34			2.03E-03	23.41	1.97E-03
29	16711680	site2	Unknown	23.57			1.84E-03		
30	12615680	site2	Unknown	23.32			2.05E-03		
31	12615680	site3	Unknown	23.71			1.71E-03	24.33	1.18E-03
32	16776960	site3	Unknown	24.88			7.06E-04		
33	16776960	site3	Unknown	24.4			1.12E-03		
34	16777088	neg	Negative Control		NEG (Multi Ct)				
35	16777088	neg	Negative Control		NEG (Multi Ct)				
36	16744576	neg	Negative Control		NEG (Multi Ct)				

Excel Analysed Data Export
Copyright (c) 2013 QIAGEN GmbH. All Rights Reserved.
File Specific Sapria 51C sampleOCT B.rex

Operator	MO	
Run Id		
Notes		
Machine Serial No		814137

Channel	Gain
Green	5
Yellow	5
Orange	5
Red	5
Crimson	7
HRM	7

Channel	Threshold
Relative Quant. Analysis	
Cycling A.Green (Page 1)	0.05574

Quantitative analysis of Cycling A.Green (Page 1)

No.	Color	Name	Type	Ct	Ct Comment	Given Conc (Copies)	Calc Conc (Copies)	% Var	Rep. Ct	Rep. Ct Std. Dev.	Rep. Ct (95% CI)	Rep. Calc. Conc.	Rep. Calc. Conc. (95% CI)
1	255	Sapria 1/10	Standard	17.04		0.1	9.91E-02		17.07			9.52E-02	
2	8388736	Sapria 1/10	Standard	16.97		0.1	1.07E-01						
3	14450322	Sapria 1/10	Standard	17.21		0.1	7.92E-02						
4	32896	Sapria 1/100	Standard	21.11		0.01	1.24E-02		21.38			1.09E-02	
5	16711680	Sapria 1/100	Standard	21.65		0.01	9.30E-03						
6	11433472	Sapria 1/100	Standard	21.39		0.01	1.08E-02						
7	11403264	Sapria 1/1000	Standard	25.01		0.001	5.94E-04		24.67			8.87E-04	

8	11338136	Sapria 1/1000	Standard	24.63	0.001	9.21E-04		
9	6553774	Sapria 1/1000	Standard	24.37	0.001	1.15E-03		
10	14013909	Sapria 1/10000	Standard	27.28	0.0001	9.62E-05	27	1.08E-04
11	12566463	Sapria 1/10000	Standard	26.99	0.0001	1.09E-04		
12	9803157	Sapria 1/10000	Standard	26.75	0.0001	1.19E-04		
13	7697781	Sapria 1/100000	Standard	28.28	0.00001	1.60E-05	28.63	1.56E-05
14	5526612	Sapria 1/100000	Standard	28.92	0.00001	1.53E-05		
15	255	Sapria 1/100000	Standard	28.69	0.00001	1.55E-05		
16	51400	Sapria tissue	Unknown	12.84		5.51E+00	13	5.34E+00
17	32768	Sapria tissue	Unknown	13.17		5.16E+00		
18	65280	Sapria tissue	Unknown	13.01		5.33E+00		
19	65280	1m	Unknown	30.02		1.40E-05	30.25	1.37E-05
20	65408	1m	Unknown	30.64		1.33E-05		
21	65408	1m	Unknown	30.1		1.39E-05		
22	8454016	5m	Unknown	32.65		1.10E-05	33.48	9.99E-06
23	8454016	5m	Unknown	34.31		9.02E-06		
24	4259584	5m	Unknown					
25	4259584	site1	Unknown	15.45		2.85E-01	15.46	2.84E-01
26	8388608	site1	Unknown	15.22		3.12E-01		
27	8388608	site1	Unknown	15.71		2.55E-01		
28	16711680	site2	Unknown	23.16		2.19E-03	23.15	2.20E-03
29	16711680	site2	Unknown	23.21		2.15E-03		
30	12615680	site2	Unknown	23.08		2.26E-03		
31	12615680	site3	Unknown	25.53		1.71E-04	25.5	1.72E-04
32	16776960	site3	Unknown	25.28		1.82E-04		
33	16776960	site3	Unknown	25.71		1.64E-04		
34	16777088	neg	Negative Control	NEG (Multi Ct)				
35	16777088	neg	Negative Control	NEG (Multi Ct)				
36	16744576	neg	Negative Control	NEG (Multi Ct)				

Excel Analysed Data Export
Copyright (c) 2013 QIAGEN GmbH. All Rights Reserved.

File	Specific Sapria 51C sampleOCT C.rex
Operator	MO
Run Id	
Notes	
Machine Serial No	814137
Channel	Gain
Green	5
Yellow	5
Orange	5
Red	5
Crimson	7
HRM	7
Channel	Threshold
Relative Quant. Analysis	
Cycling A.Green (Page 1)	0.05574

Quantitative analysis of Cycling A.Green (Page 1)

No.	Color	Name	Type	Ct	Ct Comment	Given Conc (Copies)	Calc Conc (Copies)	% Var	Rep. Ct	Rep. Ct Std. Dev.	Rep. Ct (95% CI)	Rep. Calc. Conc.	Rep. Calc. Conc. (95% CI)
1	255	Sapria 1/10	Standard	16.88		0.1	1.18E-01		16.89			1.17E-01	
2	8388736	Sapria 1/10	Standard	16.72		0.1	1.37E-01						
3	14450322	Sapria 1/10	Standard	17.07		0.1	9.56E-02						
4	32896	Sapria 1/100	Standard	22.01		0.01	7.20E-03		21.83			8.21E-03	
5	16711680	Sapria 1/100	Standard	21.84		0.01	8.19E-03						
6	11433472	Sapria 1/100	Standard	21.66		0.01	9.24E-03						
7	11403264	Sapria 1/1000	Standard	25.28		0.001	3.61E-04		25.04			5.65E-04	

8	11338136	Sapria 1/1000	Standard	24.94		0.001	6.54E-04		
9	6553774	Sapria 1/1000	Standard	24.91		0.001	6.80E-04		
10	14013909	Sapria 1/10000	Standard	27.02		0.0001	1.07E-04	26.88	1.13E-04
11	12566463	Sapria 1/10000	Standard	26.78		0.0001	1.18E-04		
12	9803157	Sapria 1/10000	Standard	26.86		0.0001	1.14E-04		
13	7697781	Sapria 1/100000	Standard	28.3		0.00001	1.60E-05	28.43	1.58E-05
14	5526612	Sapria 1/100000	Standard	28.74		0.00001	1.55E-05		
15	255	Sapria 1/100000	Standard	28.26		0.00001	1.60E-05		
16	51400	Sapria tissue	Unknown	13.08			5.26E+00	13.17	5.15E+00
17	32768	Sapria tissue	Unknown	13.33			4.99E+00		
18	65280	Sapria tissue	Unknown	13.12			5.21E+00		
19	65280	1m	Unknown	29.87			1.42E-05	30.1	1.39E-05
20	65408	1m	Unknown	30.46			1.35E-05		
21	65408	1m	Unknown	29.98			1.40E-05		
22	8454016	5m	Unknown	32.34			1.13E-05	33.43	1.00E-05
23	8454016	5m	Unknown						
24	4259584	5m	Unknown	34.53			8.77E-06		
25	4259584	site1	Unknown	15.98			2.23E-01	16.2	1.97E-01
26	8388608	site1	Unknown	16.41			1.73E-01		
27	8388608	site1	Unknown	16.22			1.95E-01		
28	16711680	site2	Unknown	23.47			1.92E-03	23.25	2.11E-03
29	16711680	site2	Unknown	23.31			2.06E-03		
30	12615680	site2	Unknown	22.98			2.34E-03		
31	12615680	site3	Unknown	26.11			1.46E-04	26.17	1.44E-04
32	16776960	site3	Unknown	26.37			1.35E-04		
33	16776960	site3	Unknown	26.03			1.50E-04		
34	16777088	neg	Negative Control		NEG (Multi Ct)				
35	16777088	neg	Negative Control		NEG (Multi Ct)				
36	16744576	neg	Negative Control		NEG (Multi Ct)				

Excel Analysed Data Export
Copyright (c) 2013 QIAGEN GmbH. All Rights Reserved.
File Specific Sapria 51C sampleAPR 1.rex

Operator MO
Run Id
Notes
Machine Serial No 814137

Channel	Gain
Green	5
Yellow	5
Orange	5
Red	5
Crimson	7
HRM	7

Channel	Threshold
Relative Quant. Analysis	
Cycling A.Green (Page 1)	0.05574

Quantitative analysis of Cycling A.Green (Page 1)

No.	Color	Name	Type	Ct	Ct Comment	Given Conc (Copies)	Calc Conc (Copies)	% Var	Rep. Ct	Rep. Ct Std. Dev.	Rep. Ct (95% CI)	Rep. Calc. Conc.	Rep. Calc. Conc. (95% CI)
1	255	Sapria 1/10	Standard	17.34		0.1	6.39E-02		17.37			6.04E-02	
2	8388736	Sapria 1/10	Standard	17.32		0.1	6.63E-02						
3	14450322	Sapria 1/10	Standard	17.45		0.1	5.10E-02						
4	32896	Sapria 1/100	Standard	22.42		0.01	4.81E-03		22.38			5.00E-03	
5	16711680	Sapria 1/100	Standard	22.63		0.01	3.58E-03						
6	11433472	Sapria 1/100	Standard	22.11		0.01	6.62E-03						
7	11403264	Sapria 1/1000	Standard	25.46		0.001	2.06E-04		25.67			1.15E-04	

8	11338136	Sapria 1/1000	Standard	25.72		0.001	1.84E-05		
9	6553774	Sapria 1/1000	Standard	25.84		0.001	1.22E-04		
10	14013909	Sapria 1/10000	Standard	27.33		0.0001	9.40E-05	27.62	8.14E-05
11	12566463	Sapria 1/10000	Standard	27.62		0.0001	8.15E-05		
12	9803157	Sapria 1/10000	Standard	27.92		0.0001	6.86E-05		
13	7697781	Sapria 1/100000	Standard	28.12		0.00001	1.60E-05	28.21	1.60E-05
14	5526612	Sapria 1/100000	Standard	28.35		0.00001	1.59E-05		
15	14013909	Sapria 1/100000	Standard	28.17		0.00001	1.61E-05		
16	12566463	Sapria tissue	Unknown	12.87			5.48E+00	13.19	5.13E+00
17	9803157	Sapria tissue	Unknown	13.28			5.04E+00		
18	7697781	Sapria tissue	Unknown	13.44			4.87E+00		
19	5526612	1m	Unknown	30.13			1.39E-05	30.45	1.35E-05
20	51400	1m	Unknown	30.79			1.31E-05		
21	16711680	1m	Unknown	30.45			1.35E-05		
22	16384	5m	Unknown	34.78			8.48E-06	34.4	8.91E-06
23	8454016	5m	Unknown						
24	4259584	5m	Unknown	34.03			9.35E-06		
25	32768	site1	Unknown	15.24			3.10E-01	15.46	2.84E-01
26	7372800	site1	Unknown	15.79			2.46E-01		
27	65280	site1	Unknown	15.36			2.96E-01		
28	16711680	site2	Unknown	23.67			1.75E-03	23.05	2.28E-03
29	8388608	site2	Unknown	23.28			2.09E-03		
30	12615680	site2	Unknown	22.2			3.02E-03		
31	16744576	site3	Unknown	25.87			1.57E-04	26.11	1.46E-04
32	16776960	site3	Unknown	26.14			1.45E-04		
33	5197647	site3	Unknown	26.33			1.37E-04		
34	16777088	neg	Negative Control		NEG (Multi Ct)				
35	8388672	neg	Negative Control		NEG (Multi Ct)				
36	16744576	neg	Negative Control		NEG (Multi Ct)				

Excel Analysed Data Export
Copyright (c) 2013 QIAGEN GmbH. All Rights Reserved.
File Specific Sapria 51C sampleAPR 2.rex

Operator	MO
Run Id	
Notes	
Machine Serial No	814137

Channel	Gain
Green	5
Yellow	5
Orange	5
Red	5
Crimson	7
HRM	7

Channel	Threshold
Relative Quant. Analysis	
Cycling A.Green (Page 1)	0.05574

Quantitative analysis of Cycling A.Green (Page 1)

No.	Color	Name	Type	Ct	Ct Comment	Given Conc (Copies)	Calc Conc (Copies)	% Var	Rep. Ct	Rep. Ct Std. Dev.	Rep. Ct (95% CI)	Rep. Calc. Conc.	Rep. Calc. Conc. (95% CI)
1	255	Sapria 1/10	Standard	16.64		0.1	9.91E-02		16.84			1.06E-01	
2	8388736	Sapria 1/10	Standard	16.87		0.1	1.19E-01						
3	14450322	Sapria 1/10	Standard	17.03		0.1	1.00E-01						
4	32896	Sapria 1/100	Standard	21.56		0.01	9.82E-03		21.69			9.06E-03	
5	16711680	Sapria 1/100	Standard	22.24		0.01	5.86E-03						
6	11433472	Sapria 1/100	Standard	21.27		0.01	1.15E-02						

7	11403264	Sapria 1/1000	Standard	25.08		0.001	5.33E-04	25.22	4.10E-04
8	11338136	Sapria 1/1000	Standard	25.48		0.001	1.88E-04		
9	6553774	Sapria 1/1000	Standard	25.11		0.001	5.07E-04		
10	14013909	Sapria 1/10000	Standard	26.47		0.0001	1.31E-04	27.05	1.06E-04
11	12566463	Sapria 1/10000	Standard	27		0.0001	1.08E-04		
12	9803157	Sapria 1/10000	Standard	27.69		0.0001	7.85E-05		
13	7697781	Sapria 1/100000	Standard	28.74		0.00001	1.55E-05	28.36	1.58E-05
14	5526612	Sapria 1/100000	Standard	28.29		0.00001	1.60E-05		
15	14013909	Sapria 1/100000	Standard	28.06		0.00001	1.60E-05		
16	12566463	Sapria tissue	Unknown	13.64			4.66E+00	13.74	4.55E+00
17	9803157	Sapria tissue	Unknown	13.41			4.90E+00		
18	7697781	Sapria tissue	Unknown	14.17			4.09E+00		
19	5526612	1m	Unknown	29.54			1.46E-05	29.68	1.44E-05
20	51400	1m	Unknown	29.33			1.48E-05		
21	16711680	1m	Unknown	30.19			1.38E-05		
22	16384	5m	Unknown						
23	8454016	5m	Unknown	34.64			8.64E-06		
24	4259584	5m	Unknown						
25	32768	site1	Unknown	16.37			1.78E-01	16.24	1.92E-01
26	7372800	site1	Unknown	16.65			1.45E-01		
27	65280	site1	Unknown	15.72			2.54E-01		
28	16711680	site2	Unknown	23			2.33E-03	23.01	2.32E-03
29	8388608	site2	Unknown	22.88			2.43E-03		
30	12615680	site2	Unknown	23.16			2.19E-03		
31	16744576	site3	Unknown	24.51			1.02E-03	24.51	1.02E-03
32	16776960	site3	Unknown	24.23			1.27E-03		
33	5197647	site3	Unknown	24.8			7.75E-04		
34	16777088	neg	Negative Control		NEG (Multi Ct)				
35	8388672	neg	Negative Control		NEG (Multi Ct)				
36	16744576	neg	Negative Control		NEG (Multi Ct)				

Excel Analysed Data Export
Copyright (c) 2013 QIAGEN GmbH. All Rights Reserved.
File Specific Sapria 51C sampleAPR 3.rex

Operator	MO
Run Id	
Notes	
Machine Serial No	814137

Channel	Gain
Green	5
Yellow	5
Orange	5
Red	5
Crimson	7
HRM	7

Channel	Threshold
Relative Quant. Analysis	
Cycling A.Green (Page 1)	0.05574

Quantitative analysis of Cycling A.Green (Page 1)

No.	Color	Name	Type	Ct	Ct Comment	Given Conc (Copies)	Calc Conc (Copies)	% Var	Rep. Ct	Rep. Ct Std. Dev.	Rep. Ct (95% CI)	Rep. Calc. Conc.	Rep. Calc. Conc. (95% CI)
1	255	Sapria 1/10	Standard	17.41		0.1	5.57E-02		17.42			5.38E-02	
2	8388736	Sapria 1/10	Standard	17.5		0.1	4.52E-02						
3	14450322	Sapria 1/10	Standard	17.37		0.1	6.04E-02						
4	32896	Sapria 1/100	Standard	21.74		0.01	8.77E-03		21.58			9.67E-03	
5	16711680	Sapria 1/100	Standard	21.68		0.01	9.12E-03						
6	11433472	Sapria 1/100	Standard	21.34		0.01	1.11E-02						

7	11403264	Sapria 1/1000	Standard	25.62		0.001	6.78E-05	25.59	1.29E-04
8	11338136	Sapria 1/1000	Standard	25.77		0.001	6.15E-05		
9	6553774	Sapria 1/1000	Standard	25.4		0.001	2.57E-04		
10	14013909	Sapria 1/10000	Standard	27.36		0.0001	9.27E-05	27.53	8.54E-05
11	12566463	Sapria 1/10000	Standard	27.41		0.0001	9.06E-05		
12	9803157	Sapria 1/10000	Standard	27.82		0.0001	7.29E-05		
13	7697781	Sapria 1/100000	Standard	28.47		0.00001	1.65E-05	28.27	1.62E-05
14	5526612	Sapria 1/100000	Standard	28.21		0.00001	1.61E-05		
15	14013909	Sapria 1/100000	Standard	28.13		0.00001	1.61E-05		
16	12566463	Sapria tissue	Standard	13.08			5.26E+00	13.17	5.15E+00
17	9803157	Sapria tissue	Standard	13.33			4.99E+00		
18	7697781	Sapria tissue	Standard	13.12			5.21E+00		
19	5526612	1m	Unknown	30.66			1.33E-05	30.58	1.33E-05
20	51400	1m	Unknown	30.13			1.39E-05		
21	16711680	1m	Unknown	30.97			1.29E-05		
22	16384	5m	Unknown	34.4			8.92E-06	33.84	4.10E+00
23	8454016	5m	Unknown						
24	4259584	5m	Unknown	33.29			8.20E+00		
25	32768	site1	Unknown	16.27			1.89E-01	16.42	1.71E-01
26	7372800	site1	Unknown	16.67			1.42E-01		
27	65280	site1	Unknown	16.34			1.81E-01		
28	16711680	site2	Unknown	22.87			2.44E-03	22.89	2.42E-03
29	8388608	site2	Unknown	23.16			2.19E-03		
30	12615680	site2	Unknown	22.64			2.64E-03		
31	16744576	site3	Unknown	25.94			1.54E-04	25.8	1.59E-04
32	16776960	site3	Unknown	25.38			1.78E-04		
33	5197647	site3	Unknown	26.1			1.47E-04		
34	16777088	neg	Negative Control		NEG (Multi Ct)				
35	8388672	neg	Negative Control		NEG (Multi Ct)				
36	16744576	neg	Negative Control		NEG (Multi Ct)				
